# Analysis of gastric electrical rhythm in patients with type 2 diabetes mellitus

**DOI:** 10.1007/s12020-024-03908-y

**Published:** 2024-06-11

**Authors:** Xixi Wang, Lianhua Ma, Miao Jiang, Hong Zhu, Chendong Ni, Xiaohua Yang, Ji Hu, Hong-Hong Zhang

**Affiliations:** 1 The Affiliated Suqian Hospital of Xuzhou Medical University, Suqian, China; 2 Department of Endocrinology, The Second Affiliated Hospital of Soochow University, Suzhou, China; 3 NO.2 People’s Hospital of Fuyang City, Fuyang, China; 4 Hai’an People’s Hospital, Nantong, China; 5 Clinical Research Center of Neurological Disease, The Second Affiliated Hospital of Soochow University, Suzhou, China

**Keywords:** Type 2 diabetes mellitus, Gastric electrical rhythm, Electrogastrogram, Diabetic complications, Diabetic gastroparesis

## Abstract

**Aim:**

To analysis the change of electrogastrogram (EGG) in patients with type 2 diabetes mellitus (T2DM), and evaluate the prevalence of abnormal gastric electrical rhythm (AGER) and its relative influencing factors.

**Methods:**

A total of 65 patients with T2DM hospitalized at the Second Affiliated Hospital of Soochow University from Dec. 2020 to Dec. 2021 were included in the cross-sectional study. General information, clinical data, and medical history data of all study subjects, including name, gender, body mass index (BMI), duration of diabetes, anti-diabetic therapies, high blood pressure (HBP) history, smoking history, and medication history, were completely collected. The results of laboratory tests, including biochemical parameters, glycosylated hemoglobin (HbA1c), fasting C-peptide, 2 h postprandial C-peptide, 24 h urine total protein (24 hUTP), urine microalbumin creatinine ratio (UACR) and estimated glomerular filtration rate (eGFR) were recorded. EGG, Gastroparesis Cardinal Symptom Index (GCSI), gastric emptying ultrasound, fundus examination, carotid artery ultrasonography, cardiac autonomic function test, heart rate variability (HRV) were all examined and recorded as well. According to the results of EGG, the subjects were divided into normal gastric electrical rhythm (NGER) group and abnormal gastric electrical rhythm (AGER) group.

**Results:**

(1) Fasting blood glucose (FBG), HbA1c, the presence of diabetic peripheral neuropathy (DPN) and diabetic cardiac autonomic neuropathy (DCAN) were significantly higher in the AGER group (*p* < 0.05). Low frequency (LF) and high frequency (HF), the indicators of HRV, were significantly lower in the AGER group (*p* < 0.05). In addition, the prevalence of feeling excessively full after meals, loss of appetite, and stomach or belly visibly larger after meals of gastrointestinal symptoms of gastroparesis were significantly higher in the AGER group (*p* < 0.05). Multiple logistic regression analysis showed that FBG and the prevalence of DCAN were the independent risk factors.

**Conclusion:**

AGER was associated with high FBG and the presence of DCAN. EGG examination is recommended for patients with gastrointestinal symptoms and clues of DCAN.

## Introduction

According to the latest International Diabetes Federation report, the number of diabetic patients is more than 1.4 million in China [1]. The harm of diabetes mainly comes from its complications, including diabetic retinopathy (DR), diabetic nephropathy (DN), diabetic peripheral neuropathy (DPN), diabetic autonomic neuropathy (DAN), diabetes arteriosclerosis (DAS), etc [2]. In recent years, diabetic autonomic neuropathy characterized by gastric motility disorders (GMD) has attracted more and more attention, now that an increasing amount of evidence showed that gastrointestinal symptoms increased in diabetic patients [3]. Diabetic gastrointestinal autonomic neuropathy can eventually progress to diabetic gastroparesis (DGP) characterized by delayed gastric emptying and upper gastrointestinal symptoms. DGP can further lead to obvious blood glucose fluctuations, repeated hypoglycemia, decreased drug absorption rate, poor nutritional status, increased hospitalization rate, severe decline in quality of life, and even death [4]. Patients with diabetic gastric motility disorders (DGMD) are mainly manifested by nausea, early satiation, and abdominal distention [5]. However, Some DGMD patients only show uncontrollable postprandial hyperglycemia and recurrent hypoglycemia. The symptoms of DGMD are sometimes not so obvious that many patients are diagnosed when DGP has occurred [6]. Early detection, diagnosis, and treatment of DGMD not only delay the progress but also improve the control of diabetes and its chronic complications.

Gastric emptying scintigraphy (GES) for measuring gastric emptying is the “gold standard” for the diagnosis of DGP. However, it can not be widely used because of its radioactivity, high cost, and complex operation. Many domestic hospitals lack effective means to evaluate DGMD. Electrogastrogram (EGG) is a non-invasive method of recording gastric electrical signals through the body surface and then indirectly reflecting changes in gastric motility [7]. EGG can provide a wealth of information about the electrical activity of the gastric smooth muscle, such as gastric electrical frequency, regularity, amplitude, and power spectrum. Due to the simple operation and low price of EGG, it is widely recognized and applied. In addition, Gastroparesis Cardinal Symptom Index (GCSI) can also be used to evaluate DGMD. GCSI can be scored for the clinical symptoms of gastroparesis with the advantage of simplicity, effectiveness, authenticity, and reliability.

The purpose of this study is to analyze the change of EGG in T2DM patients and explore the prevalence of abnormal gastric electrical rhythm (AGER) in T2DM as well as the related influencing factors of AGER.

## Research design and methods

### Research design

According to the criteria for admission, the clinical data, laboratory tests, and blood samples of T2DM patients were collected and analyzed. We divided the patients into the normal gastric electrical rhythm (NGER) group and the AEGR group according to the results of EGG.

### Study protocol

A total of 65 T2DM patients with good cognitive function and ages ranging from 18 to 75 were recruited from the endocrine department of the Second Affiliated Hospital of Soochow University. The diagnosis of T2DM was based on the diagnostic criteria of WHO in 1999. The exclusion criteria are as follows: 1. pregnant or lactating female patients; 2. patients with previous gastric surgery, serious digestive system diseases, systemic diseases affecting digestive function (such as gastric cancer, gastric ulcer, pancreatic and biliary diseases), or systemic diseases affecting digestive function (such as hyperthyroidism and hypothyroidism) or electrolyte disorders; 3. used drugs affecting gastric emptying and autonomic nervous function in the past 2 weeks; 4. patients with acute complications such as hypertonic hyperglycemia state (HHS), diabetic ketoacidosis (DKA), severe hepatic and renal insufficiency (elevated transaminases more than 2 times normal, eGFR < 45 ml/min/1.73 m^2^). 5. neurological damage caused by other reasons, such as stroke, Guillain Barre syndrome, connective tissue disease, etc. 6. Patients with severe cataract and unable to complete fundus examination. During the interviews, physicians or the coordinators completed case report forms, recording the disease profiles of diabetes and related treatment histories. The protocol of the present study was approved by the Institutional Review Board of the Second Affiliated Hospital of Soochow University, and the approving registration number is JD-HG-2022-07. The patients’ personal privacy was closely guarded: the sample data was stored electronically in a special computer with a password, which was only available to researchers. The patient’s medical records were kept in the hospital.

### Clinical and biochemical measurements

Each patient received anthropometric measurements, including body weight, height, measured in light clothes and bare feet, and blood pressure (BP). Hypertension was defined as blood pressure measurement ≥140/90 mmHg in resting state or subjects with diagnosed hypertension before and taking antihypertensive drugs at present. If the patient has previously been diagnosed with hypertension and is currently receiving antihypertensive medication, he or she will still be considered as hypertension patient even if the measured blood pressure is less than 140/90 mmHg. Body mass index (BMI) was calculated according to the formula: BMI (kg/m²) = weight (kg)/ height (m)².

Any medication that interferes with gastric myoelectric activity was discontinued 48 h before EGG was performed such as metoclopramide, dmopridone, mosapride, antispasmodic, anticholinergic drug, proton pump inhibitors, glp-1 receptor agonizts etc. Ask the patient to lie down and acquire the original waveform for 30 min in the patient’s fasting state. Then the patient was asked to sit and had a test meal (five pieces of Daliyuan French buns, with a total weight of 100 g and energy content of 1422 kJ, 1 ham sausage, with a total weight of 60 g and energy content of 435 kJ, and water of less than 400 ml) within 5 min. Then patient was asked to lie down and continue to collect the original waveform for 30 minutes after the meal. Use the EGG analysis software to analyze the data automatically. Power ratio (PR) reflects the power change of EGG before and after the meal. The PR value of healthy adults should be greater than 1, otherwise it will prompt the gastric motility disorder. According to the results of EGG, we divided patients into NGER group with PR < 1 and AGER group with PR ≥ 1.

GCSI is a subset of questions from the Patient Assessment of Upper Gastrointestinal Symptom Severity Index (PAGI-SYM) questionnaire that is used to assess symptom severity in gastroparesis patients. The GCSI consists of 3 subset of questions including bloating (2 questions), nausea (3 questions), and fullness (4 questions) with total of 9 questions. All symptoms are assessed on a scale of none (0) to extremely severe (5), with the GCSI score being the average of the three subscales [8]. Patients with GCSI score ≥1.9 were considered to have gastroparesis symptoms [9].

Cardiovascular autonomic function was tested using the Medeia 3000 (China MedKinetic). The Ewing test was used to evaluate heart rate and blood pressure responses to postural change. Four parameters were evaluated, including heart rate differences caused by deep breathing, Valsalva action, and horizontal-vertical conversion, as well as blood pressure differences caused by horizontal-vertical conversion. The Ewing score was used to diagnose CAN [10]. HRV was also monitored. The time and frequency domains were generated automatically using Medeia 3000 including high frequency (HF), low frequency (LF), SDNN, and PNN50 [11]. Carotid plaque detection was tested by the Korea GE Color Ultrasound Diagnostic Instrument (LOGIQ S7). Diagnosis of atherosclerosis was according to the 2009 Chinese Physicians Association Ultrasound Division Vascular Ultrasound Guidelines. The diagnostic criteria for atherosclerotic plaque are that the carotid intimal thickening exceeds 50% of the peripheral IMT or protrudes into the vascular lumen, and IMT ≥ 1.5 mm [12]. The diagnosis of DN refers to the 《2020 Expert Consensus on diagnosis and treatment of diabetic nephropathy》. The diagnosis of DR refers to the DR grading standards established by the International Society of Ophthalmology in 2002. The diagnosis of DPN refers to the clinical diagnostic criteria recommended by the 2009 Toronto International Conference on Diabetic Peripheral Neuropathy [13].

All patients fasted for at least 8 h before blood samples were collected. The fully automated blood cell analyzer (Sysmex kx-21N, Japan) was used to determine blood routines. Biochemical parameters, such as fasting blood glucose (FBG), triglyceride (TG), total cholesterol (TC), high-density lipoprotein (HDL-C), low-density lipoprotein (LDL-C), uric acid (UA), creatinine (Cr), blood urea nitrogen (BUN), serum albumin (Alb), C-reactive protein (CRP) and urinary microalbumin creatinine ratio (UACR) were measured with an automated biochemical instrument (Cobas800-c702, Roche, Basel, Switzerland). Roche Cobas e601 (Roche Diagnostic, Germany) was used for fasting C-peptides and 2 h postprandial C-peptides. The Bole D100 glycation hemoglobin meter (Bole, USA) was used for hemoglobin A1c (HbA1c). eGFR was calculated with the modified MDRD equation.

### Statistical methods

Software SPSS 24.0 was used for data analysis. The single sample K-S test was used to check whether the data conform to the normal distribution. Data conforming to normal distribution in continuous variables were presented as means ± standard deviation (SD), while non-normal distribution data were presented by median(IQR), and categorical variables are presented as frequency (n) or percentage (%). Comparisons between different groups were tested using two-sample *t*-test, Mann-Whitney *U* test, or *χ*2 test. Logistic regression analysis was used to evaluate the risk factors of NEGR. A *p* value less than 0.05 was considered to be statistically significant.

## Results

### A total of 65 patients with type 2 diabetes mellitus were enrolled in the study

Of the 65 diabetic patients, average age was 53.60 years with 36.92% being females, and average BMI was 24.14 kg/m^2^.

### The results of EGG showed that 16 patients had AGER with a prevalence of 24.62%

The average proportion of slow waves before and after meals was less than 65%, and postprandial bradykinesia was the main symptom (Table 1).

**Table 1:** EGG results in patients with type 2 diabetes

EGG parameters	Proportions (%)
Average pre-meal slow-wave ratio	54.1
Average postprandial slow wave ratio	48.9
Average proportion of pre-meal delays	20.4
Average proportion of postprandial delays	24.5
Average ratio of overspeed before a meal	17.6
Average postprandial overspeed ratio	22.2

As shown in Table 2, the patients were divided into the AGER group and the NGER group. FBG, HbA1c were significantly higher in the AGER group (**p < 0.01, *p < 0.05, compared with NGER, two-sample *t*-test). LF and HF were much lower in the AGER group (**p < 0.01, *p < 0.05, compared with NGER, two-sample *t*-test). The presence of AGER was associated with the presence of DPN and DCAN (Table 2, **p < 0.01, *p < 0.05, *χ*2 test). In the logistic regression analysis, we set the presence or absence of AGER as a dependent variable and the other six variables as independent variables. The findings revealed that FBG and DCAN were still independently associated with the risk of AGER (Table 3, **p < 0.01, *p < 0.05), while HbA1c, LF, HF, and DPN were no longer related.

**Table 2:** Clinical and biochemical characteristics of the AGER group and NGER group

Variables	NGER (n = 49)	AGER (n = 16)	*p* Value
Gender (M:F)	65.3% vs. 34.7%	56.3% vs. 43.7%	0.515
Age (year)	54.120 ± 10.925	52.000 ± 13.206	0.485
Duration (year)	7 (1, 10.5)	3 (1, 10)	0.430
BMI (kg/m^2^)	24.358 ± 3.777	23.489 ± 2.607	0.396
FPG (mmol/L)	8.958 ± 3.184	13.649 ± 3.984	0.000**
Fasting C-peptide (ng/ml)	1.650 ± 1.134	1.619 ± 1.364	0.931
C-peptide 2 h postprandial (ng/ml)	2.740 (1.750, 5.370)	3.130 (2.110, 6.320)	0.572
HbA1c (%)	9.138 ± 2.055	11.794 ± 1.952	0.000**
TG (mmol/L)	2.489 ± 2.381	1.534 ± 1.133	0.055
TC (mmol/L)	4.618 ± 1.014	4.774 ± 1.623	0.652
LDL-C (mmol/L)	2.780 ± 0.887	3.036 ± 1.335	0.392
HDL-C (mmol/L)	1.073 ± 0.306	1.210 ± 0.381	0.155
Cr (umol/L)	58.000 (51.000, 72.000)	62.500 (48.250,72.750)	0.899
UA (umol/L)	310.380 ± 107.601	347.810 ± 125.682	0.254
BUN (mmol/L)	6.209 ± 2.419	7.681 ± 5.386	0.139
eGFR (ml/min/1.73m^2^)	103.831 ± 15.374	105.476 ± 30.887	0.781
UACR (mg/g)	14.000 (8.000, 43.000)	16.000 (10.000, 451.500)	0.600
CRP (mg/L)	5.400 (1.450, 6.200)	5.450 (3.525, 5.700)	0.605
Alb (g/L)	41.227 ± 4.716	42.894 ± 4.630	0.227
24 h-UTP (g/24 h urine)	0.040 (0.030, 0.150)	0.050 (0.025, 0.090)	0.870
LF (Hz)	178.491 ± 47.577	64.320 ± 30.228	0.024*
HF (Hz)	148.492 ± 29.367	57.013 ± 36.905	0.006**
SDNN (ms)	29.270 ± 16.332	22.430 ± 5.907	0.075
DR (Y: N)	22.4% vs. 77.6%	12.5% vs. 87.5%	0.388
DPN (Y: N)	20.0% vs. 80.0%	70.0% vs. 30.0%	0.000^**^
DN (Y: N)	23.4% vs. 76.6%	23.1% vs. 76.9%	0.699
DCAN (Y: N)	13.2% vs. 86.8%	53.8% vs. 46.2%	0.000^**^
CP (Y: N)	61.4% vs. 38.6%	38.5% vs. 61.5%	0.144
Smoke (Y: N)	30.6% vs. 69.4%	25.0% vs. 75.0%	0.668
HBP (Y: N)	44.9% vs. 55.1%	43.8% vs. 56.2%	0.936
Insulin therapy (Y:N)	44.9% vs. 55.1%	60.0% vs. 40.0%	0.604
Metformin therapy (Y:N)	36.7% vs. 63.3%	43.8% vs. 56.2%	0.617

Data are means ± SD or medians (interquartile ranges) or numbers (percentage) of patients. *p* values for differences between two groups were obtained by two-sample *t*-test, Mann-Whitney U- test, or χ2 test

*BMI* body mass index, *FPG* fasting plasma glucose, *HbA1c* glycated hemoglobin A1c, *TG* triglycerides, *TC* total cholesterol, *LDL-c* low density lipoprotein cholesterol, *HDL-c* high density lipoprotein cholesterol, *Cr* creatinine, *UA* uric acid, *BUN* blood urea nitrogen, *eGFR* estimated glomerular filtration rate, *UACR* urinary albumin creatinine ratio, *CRP* c-reactive protein, *Alb* albumin, *24* *h-UTP* 24 hour urine total protein, *LF* low frequency, *HF* high frequency, *SDNN* standard deviation of NN intervals, *DR* diabetic retinopathy, *DPN* diabetic peripheral neuropathy, *DN* diabetic nephropathy, *DCAN* diabetic cardiac autonomic neuropathy, *CP* carotid plaque, *HBP* high blood pressure

**p < 0.01, *p < 0.05

**Table 3:** AGER as dependent variable in multiple logistic regression analysis

Variables	β	OR (95%CI)	*p* Value
FBG	0.529	1.608 (1.192, 2.169)	0.004**
DCAN	2.263	14.342 (1.294, 159.026)	0.030*
HbA1c	0.571	1.770 (0.615, 5.097)	0.153
DPN	1.530	4.618 (0.096, 221.357)	0.234
LF	−0.002	0.998 (0.985, 1.011)	0.659
HF	−0.001	0.999 (0.980, 1.018)	0.492

In multiple logistic regression analysis, diabetic gastric motility disorder, as dependent variable, and the other 6 variables, FBG, HbA1c, LF, HF, DCAN, and DPN, as independent variables, were included in the same model. Only 2 variables, FBG, and DCAN were the risk factors of AGER

**p < 0.01, *p < 0.05

### A total of 65 T2DM patients completed GCSI questionnaire

Of the 65 patients, 33 (50.77%) patients had at least one symptom of gastrointestinal comfort. Among all gastrointestinal symptoms, “reduced appetite” was the most common, with a prevalence of 47.7% (31/65), while “vomiting” was the least common, with a prevalence of 1.5% (1/65). The prevalence of gastrointestinal symptoms was detailed in Table 4.

**Table 4:** Prevalence of gastrointestinal symptoms of gastroparesis in patients with Type 2 diabetes

Items	Men [n (%)]	Women [n (%)]	Total [n (%)]
Nausea	11 (26.8)	10 (41.7)	21 (32.3)
Retching	11 (26.8)	10 (41.7)	21 (32.3)
Vomiting	1 (2.4)	0 (0.0)	1 (1.5)
Stomach fullness	12 (29.3)	15 (62.5)	27 (41.5)
Inability to finish a normal-sized meal	15 (36.6)	16 (66.7)	31 (47.7)
Feeling excessively full after meals	12 (29.3)	14 (58.3)	26 (40.0)
Loss of appetite	12 (29.3)	14 (58.3)	26 (40.0)
Bloating	14 (34.1)	15 (62.5)	29 (44.6)
Stomach or belly visibly larger after meals	11 (26.8)	14 (58.3)	25 (43.1)

As shown in Table 5, the patients were divided into the AGER group and the NGER group. The prevalence of feeling excessively full after meals, loss of appetite, and stomach or belly visibly larger after meals of gastrointestinal symptoms of gastroparesis were significantly higher in the AGER group. (Table 5, *p < 0.05, *χ*2 test).

**Table 5:** Prevalence of gastrointestinal symptoms of gastroparesis of the AGER group and NGER group

Variables	NGER (n = 49)	AGER(n = 16)	*p* Value
Nausea	28.57%	43.75%	0.260
Retching	28.57%	43.75%	0.260
Vomiting	0%	6.67%	0.078
Stomach fullness	34.69%	62.5%	0.050
Inability to finish a normal-sized meal	42.86%	62.5%	0.172
Feeling excessively full after meals	48.48%	62.5%	0.034*
Loss of appetite	48.48%	62.5%	0.034*
Bloating	38.78%	62.5%	0.097
Stomach or belly visibly larger after meals	30.61%	62.5%	0.023*

Data are the percentage of patients. *p* values for differences between two groups were obtained by χ2 test

*p < 0.05

## Discussion

Diabetes mellitus is a chronic metabolic disease which can affect multiple systems and lead to devastating complications. DGP is one of its chronic complications of diabetes, which refers to the presence of chronic gastric motor dysfunction and delayed gastric emptying in the absence of mechanical obstruction of the gastrointestinal tract, and its main pathological basis is GMD [14]. There are no overt symptoms in the early stage of DGP. As the condition worsens, some gastrointestinal symptoms such as nausea, vomiting, and epigastric fullness appear alone or together [15]. In many cases, diabetes patients’ gastrointestinal symptoms may not always accurately reflect their gastric motility status, necessitating the use of objective measures to assess gastrointestinal function [16]. Gastric emptying scintigraphy (GES), as the “gold standard” for gastric motility testing, is limited used because of its high cost, expensive equipment, and radiation. The myoelectric activity of the stomach controls the contractile activity, and the patients with GMD often have abnormal gastric electric rhythms. EGG, as a non-invasive method of recording gastric electrical signals through the body surface, can indirectly reflect gastric motility changes and has the advantage of being less expensive and easy to operate compared to GES. Besides, it has no stimulation to the gastrointestinal tract and can observe gastric electrical activity for a long time. EGG is considered to be an ideal examination method for the screening of DGP with important clinical application value [17]. EGG is of great significance for the diagnosis, severity evaluation, and treatment guidance of patients. In the present study, we applied EGG to evaluate gastric motility in patients with T2DM and found that the T2DM patients generally suffered from gastric electrical rhythm disorder and decreased gastric motility. The analysis of influencing factors showed that FBG, HbA1c were significantly higher in the AGER group, and the presence of AGER was associated with the presence of DPN and DCAN.

High FBG reflects immediate hyperglycemia, and high HbA1c reflects the average high blood glucose in the recent 3 months which means chronic hyperglycemia. Acute changes in blood glucose concentration affect gastric emptying in patients with diabetes [18]. Gastric emptying is slower during hyperglycemia and accelerated during hypoglycemia. This response is a physiological defense mechanism to prevent further hyperglycemia and hypoglycemia [19]. Under acute hyperglycemia conditions, gastric emptying can be slowed down through a series of combined actions, including inhibited vagus nerve activity and proximal gastric tension, reduced antral pressure, and stimulated pyloric contraction [20, 21]. This conclusion was also confirmed in healthy people that acute hyperglycemia inhibited gastric antrum movement and induced abnormal gastric rhythm in healthy volunteers [22]. Chronic hyperglycemia can also cause GMD, which was concluded from several physiological studies on diabetic gastrointestinal function [21, 23]. Studies have found that chronic hyperglycemia leads to the formation of advanced glycation end products (AGEs), excessive oxidative stress, inflammatory activation, through a series of pathways, which damage the structure of nerve cells, reduce the number of neurons, and lead to degeneration of the nervous system [24]. In addition, it also reduces the number of interstitial cells of Cajal (ICC) and damages gastric smooth muscle cells, resulting in decreased contractility and ultimately delayed gastric emptying [14, 24].

On the other hand, gastric emptying also has an impact on the level of blood glucose, and delayed gastric emptying affects the absorption of food, which directly affects the blood glucose level after a meal. DGMD can also lead to poor absorption of drugs, which in turn has an adverse effect on blood glucose control. For patients who use insulin, the effect of exogenous insulin on the body does not match the digestion and absorption of food in the gastrointestinal tract caused by DGMD [25]. It is very easy to have large fluctuations of postprandial blood glucose including early postprandial hypoglycemia and late postprandial hyperglycemia [26]. The onset of hypoglycemia will cause irreversible damage to the human body, which must be taken seriously. In clinical practice, the possibility of DGMD should be considered in diabetic patients with poor blood glucose control after repeated adjustment of treatment regimens who encounter repeated hypoglycemia after meals and large fluctuations in postprandial blood glucose.

Diabetic autonomic neuropathy is currently recognized as one of the main pathophysiological foundations of DGMD [14, 15]. The autonomic nervous system includes sympathetic and parasympathetic nerves that innervate multiple organs, including cardiovascular, gastrointestinal, respiratory, urogenital, and pupillary. The sympathetic and parasympathetic nerves of T2DM patients are damaged to varying degrees, resulting in cardiac autonomic neuropathy, gastrointestinal autonomic neuropathy, etc [27]. In the present study, we found that DCAN was independently associated with AGER, and there was a strong association between HRV and AGER. HRV is a good indicator of cardiac autonomic balance. Growing studies supported the use of HRV to assess cardiac autonomic activity. We also found that LF and HF, two HRV indicators, were significantly lower in the AGER group, indicating that AGER patients had both sympathetic and vagus nerve damages, which was in line with prior research findings [28, 29]. In clinical practice, autonomic neuropathy is often underdiagnosed and poorly treated. More attention should be paid to diabetic autonomic neuropathy [30].

For the evaluation of severity of symptoms in DGP patients, GCSI is considered an effective, non-invasive, inexpensive assessment tool for epidemiological screening. There is a large number of studies supporting the reliability of the GCSI scoring scale [31]. In the present study, AGER, detected by EGG, correlated with upper gastrointestinal symptoms. As expected, subjects in AGER group had more severe symptoms compared with NGER group subjects. Patients in AGER group were more likely to experience feeling excessively full after meals, loss of appetite, stomach or belly visibly larger after meals. As an object measure that correlates with symptoms, EGG can be used widely in clinics to evaluate gastric disease progression and the effectiveness of treatment.

Certain oral hypoglycemic drugs may cause gastrointestinal symptoms in patients with diabetes. Studies have shown that metformin treatment was significantly associated with gastrointestinal symptoms in DGP patients [32]. However, we didn’t get the same conclusion in the present study. The average course of diabetes in this study was 7 years, patients who were still using metformin may have adapted to the drug, or they may have switched to other anti-diabetic regimens if they have any severe gastrointestinal symptoms. This may explain our failure to detect a causal link between metformin and gastrointestinal symptoms. Glucagon-like peptides receptor agonizts (GLP-1RA) may also cause gastrointestinal symptoms in diabetic patients. However, patients using GLP-1 receptor agonizts were excluded from this study. In clinical practice, it is recommended that patients who are ready to use GLP-1RA have their gastrointestinal symptoms be evaluated, using GCSI, EGG, and gastric emptying tests, to avoid obvious gastrointestinal side effects.

## Conclusion

EGG effectively reflects gastric electrical signals and can indirectly assess gastric motility status. AGER in T2DM patients was associated with high FBG and the presence of DCAN.

## Abbreviation

EGG: electrogastrogram
AGER: abnormal gastric electrical rhythm
NGER: normal gastric electrical rhythm
GMD: gastric motility disorder
GCSI: Gastroparesis Cardinal Symptom Index
DGMD: Diabetic gastric motility disorder
HbA1c: Glycated hemoglobin A1c
24-hUTP: 24 hour urine total protein
UACR: Urine microalbumin creatinine ratio
eGFR: Estimated glomerular filtration rate
HRV: Heart rate variability
LF: low frequency
HF: high frequency
DPN: Diabetic peripheral neuropathy
DCAN: Diabetic cardiac autonomic neuropathy
DR: Diabetic Retinopathy
DN: Diabetic Nephropathy
DAN: Diabetic autonomic neuropathy
DGP: Diabetic gastroparesis
GLP-1: Glucagon-like peptides
GES: Gastric emptying scintigraphy
HHS: hyperglycemic hyperosmolar syndrome
DKA: diabetic ketoacidosis
PR: power ratio
ECG: electrocardiogram
CP: Carotid plaque
c-IMT: carotid intima-media thickness
ICC: Interstitial cells of Cajal.
